# Maternal immunization against Group B streptococcus: World Health Organization research and development technological roadmap and preferred product characteristics

**DOI:** 10.1016/j.vaccine.2017.09.087

**Published:** 2019-11-28

**Authors:** Johan Vekemans, Vasee Moorthy, Martin Friede, Mark R. Alderson, Ajoke Sobanjo-Ter Meulen, Carol J. Baker, Paul T. Heath, Shabir A. Madhi, Kirsty Mehring-Le Doare, Samir K. Saha, Stephanie Schrag, David C. Kaslow

**Affiliations:** a World Health Organisation, Geneva, Switzerland; b PATH, Seattle, USA; c Bill & Melinda Gates Foundation, Seattle, USA; d Baylor College of Medicine, Houston, USA; e Vaccine Institute, St Georges, University of London, London, UK; f Medical Research Council: Respiratory and Meningeal Pathogens Research Unit, University of the Witwatersrand, Johannesburg, South Africa; g Imperial College Faculty of Medicine, London, UK; h Child Health Research Foundation, Dhaka Shishu Hospital, Institute of Child Health, Dhaka, Bangladesh; i Centers for Disease Control and Prevention, Atlanta, USA

**Keywords:** Group B streptococcus, Vaccine, Maternal immunization

## Abstract

Group B streptococcus, found in the vagina or lower gastrointestinal tract of about 10–40% of women of reproductive age, is a leading cause of early life invasive bacterial disease, potentially amenable to prevention through maternal immunization during pregnancy. Following a consultation process with global stakeholders, the World Health Organization is herein proposing priority research and development pathways and preferred product characteristics for GBS vaccines, with the aim to facilitate and accelerate vaccine licensure, policy recommendation for wide scale use and implementation.

It is now over forty years ago that an association between transplacental transfer of maternal Group B streptococcus (GBS) antibodies and infant protection from invasive infection was shown, supporting the concept of a maternal immunization-based prevention strategy [1]. GBS, an encapsulated bacterium found in the vagina or lower gastrointestinal tract of about 10–40% of women of reproductive age, is a leading cause of neonatal and infant invasive bacterial disease, often leading to death or neurological sequelae. Ascending infections during pregnancy can lead to stillbirths and premature delivery, and GBS can cause puerperal sepsis and other maternal morbidities [2]. Carriage or risk factor-based screening followed by intrapartum antibiotic prophylaxis (IAP) can reduce the risk of GBS disease in the first week of life, but this approach has only been partially successful. It is ineffective in preventing late-onset GBS infant disease, is associated with a substantial amount of perinatal antibiotic use, and has not been implemented on a systematic basis in most low- and middle-income countries. There is a significant residual disease burden in high-income countries [3], [4].

Past investments in GBS vaccine research have been limited, probably due to a (mis)perception that IAP based strategies are sufficient to deal with the disease in high-income countries, concerns about the complexity of developing vaccines for use in pregnancy, and an incomplete evidence base on the global disease burden. New data, a better understanding of the limitations of existing control strategies and progress in the definition of regulatory and policy pathways for immunization during pregnancy have renewed the interest in vaccine strategies against perinatal GBS disease [5], [6]. Considering the available evidence about the role of passively transferred GBS antibodies from the mother to the neonate, the technical feasibility of developing a GBS vaccine is estimated to be high [7], [8]. In line with its mission to provide guidance on research and development pathways targeting diseases of high public health interest, the World Health Organisation has just made two technical documents publically available: a GBS vaccine development technology roadmap presenting a priority action framework, and preferred product characteristics [9]. The aim is to facilitate and accelerate vaccine development and guide the work of researchers, industry and funders with respect to clinical development data collection requirements, ensuring that critical, relevant public health questions are answered. The intent of this work is to support robust policy decision-making for licensed products to be practically implemented where most needed, without undue delays.

As expressed in these documents, the strategic goal is to see the development and licensure of safe, effective and affordable GBS vaccines for maternal immunization during the second or third trimester of pregnancy to prevent GBS-related stillbirth and invasive GBS disease in neonates and young infants, appropriate for use in high-, middle- and low-income countries. A target of 80% protection against the combined risk of laboratory-confirmed GBS stillbirth and invasive disease in the offspring was set.

Among the research priorities, more and better data are needed to quantify the precise potential public health impact of a GBS vaccine. Very little information is available from some of the poorest world regions. The vaccine composition will need to overcome the diversity of bacterial capsular types or target protein expression prevalence and polymorphism, targeting at least 90% of the current invasive disease isolates. Long-term strain composition monitoring should be planned and the potential for capsular switching, strain evolution and replacement of the bacterial population as a cause of invasive disease considered. In line with principles of safety precaution, there is a preference for an adjuvant-free formulation, although the inclusion of an aluminium salt or another adjuvant with an extensively demonstrated favourable safety profile in pregnancy would likely be acceptable.

Detailed determinants of immunogenicity in pregnant women and antibody transfer to offspring should be characterized. There is a clear preference for a one-dose regimen. The role of past natural GBS exposure, of a priming dose, of boosters during subsequent pregnancies will need to be defined. Immunogenicity upon co-administration with recommended vaccines for use in pregnancy, and impact on immune responses to relevant infant vaccines (considering both the target antigen and potential presence of similar protein carriers) need to be evaluated.

The development of a quality-assured, regulatory-acceptable immune correlate of protection could accelerate and would reduce the cost of vaccine development. Further assay development, detailed immuno-epidemiological and vaccine studies are needed to confirm whether, in addition to favourable safety being established, large pre-licensure efficacy trials are needed, or whether it would be acceptable for licensure and policy decision to be based on indirect evidence of efficacy, with more comprehensive health impact evaluation ongoing after initial licensure, such as during pilot implementation projects.

In the absence of evidence that supports licensure based on immune correlates of protection, a pivotal efficacy trial will be needed. Endpoint case definitions and ascertainment methodologies will need to be defined and standardized, including in the case of stillbirth or fatality. Priority endpoints are presented. Leading principles about appropriate standards of care for maternal and infant infectious risk management in the context of a GBS vaccine efficacy trial are proposed, in due consideration of local standards of care and WHO recommendations on infection risk management.

The influence of important maternal comorbidities such as HIV infection and malaria in pregnancy on vaccine outcomes should be evaluated. Considering the high prevalence of HIV infection in certain regions with high GBS disease incidence, and the much increased risk of invasive GBS disease in HIV-exposed infants, HIV should not be a contra-indication to vaccination.

Robust clinical development planning, data collection and delivery will require constitution of networks of investigators, including research centres in low- and middle-income countries with strong clinical trial research capacity, documented baseline rates of disease and of common adverse obstetric and neonatal outcomes, and appropriate regulatory and ethical oversight. Global recommendations and resources are available to support adequate assessment of the safety of vaccines for use in pregnancy. The maternal immunization delivery platform needs to be rationalized based on a long-term vision, considering the various disease targets and the need for integrated prenatal care.

The cost of research, production, procurement and delivery must be manageable and ensure that when available, there are no access barriers for products that meet critical medical needs, and no time gap between low- and high-income countries. The residual disease burden in high-income countries should be re-assessed, with due consideration of adverse consequences of IAP-associated perinatal antibiotic use. Precise estimates of the global health economic value of resources and efforts spent on GBS vaccine development should guide responsible public and private investments. Funding partnerships may be required. In terms of approval and implementation, the WHO prequalification and SAGE policy recommendation pathway, complementary to national regulatory licensure, provide a way to ensure programmatic suitability and constitute a requirement for some financing and procurement agencies.

## Ethics committee approval

Not applicable.

## Role of the funding source

The funding source had no role in the development of the present manuscript.
